# A new and versatile template towards vertically oriented nanopillars and nanotubes†

**DOI:** 10.1039/d3na00476g

**Published:** 2023-08-09

**Authors:** Bohao Xu, Di Wu, Ian M. Hill, Merissa Halim, Yves Rubin, Yue Wang

**Affiliations:** a Department of Materials Science and Engineering, University of California Merced USA yuewang@ucmerced.edu; b Department of Chemistry and Biochemistry, University of California Los Angeles USA; c Department of Chemistry and Biochemistry, University of California Merced USA

## Abstract

Vertically oriented nanostructures bring unparalleled high surface area, light trapping capability, and high device density to electronic, optoelectronic, and energy storage devices. However, general methods to prepare such structures remain sparse and are typically based on anodized metal oxide templates. Here, we demonstrate a new approach: using vertically oriented tetraaniline nanopillar arrays as templates for creating nanopillars and nanotubes of other materials. The tetraaniline templates are scalable and easy to prepare. Vertical arrays of a variety of materials can be created by directly coating them onto the tetraaniline nanopillars *via* vapor, solution, or electrodeposition. Since the tetraaniline template is encased within the target material, it does not require post-deposition removal, thus enabling vertical structure formation of sensitive materials. Conversely, removal of the encased tetraaniline template provides vertically oriented nanotube arrays in a lost-wax-type operation. The resulting vertical structures exhibit a high degree of orientation and height uniformity, with tunable feature size, spacing, and array density. Furthermore, the deposition location and shape of the vertical arrays can be patterned at a resolution of 3 μm. Collectively, these attributes should broaden the material repertoire for vertically oriented structures, and lead to advancements in energy storage, electronics, and optoelectronics.

## Abstract 2

A direct template based on vertically oriented tetraaniline provides a new, general route towards vertically oriented nanopillar and nanotube arrays for a wide variety of materials. The arrays can also be patterned at micron-resolution.

## Graphical abstract

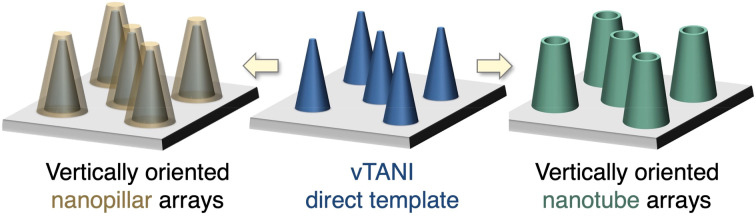

## 1. Introduction

Vertically oriented nanostructures such as nanowires, nanopillars, and nanotubes possess the distinct advantages of having high surface area, high coverage density, directional charge transport, and light trapping capability.^1–3^ These characteristics make them especially attractive for applications in electronics,^4^ optoelectronics,^5,6^ energy storage,^7,8^ bioelectronics,^9^ catalysis,^10^ and sensors.^11^ For example, the light trapping capability of vertically oriented silicon-based nanowires has led to high efficiency solar cells.^12^ The high surface area of vertically oriented nanowire or nanotube arrays has fostered the fabrication of sodium-ion batteries that exhibit high energy and power density.^13^ III–V semiconductor-based field effect transistors with high device density and high charge transport mobility have been obtained by taking advantage of the directional charge transport and high nanowire areal density associated with vertical alignment.^14,15^ Furthermore, vertical orientation of rigid and stiff materials can transform the resulting devices into flexible entities, because the bending direction of the substrate is perpendicular to the long axis of the nanostructures.^16,17^

However, vertically oriented nanostructures are challenging to produce. In general, they can be created by top-down, bottom-up, or templated approaches. Top-down methods typically involve etching through a mask, which can be prepared using self-assembled colloidal nanoparticles,^18,19^ treated block copolymers,^20^ or lithographically patterned homopolymers,^21^ ceramics,^22^ or metal films.^23^ While the top-down method is versatile for inorganic materials, it is generally not suitable for organic electronic materials because their electronic properties are easily damaged by harsh solvents or reactive ion-etching conditions. On the opposite end, bottom-up approaches can lead to vertically oriented nanostructures without a mask or etching. Chemical vapor deposition (CVD), in combination with substrate patterning or nanoparticle seeding, is the most commonly used approach.^24,25^ It offers exquisite control over diameter, height, pitch-to-pitch spacing, doping level, and crystallographic orientation of the resulting nanostructures. Unfortunately, CVD growth is material-specific and usually requires costly specialized equipment.

The most universal method for fabricating vertically oriented structures uses an inverse-templating approach. Such templates possess the negative structure of the desired morphology. Material of interest is deposited into the vacant space. Upon the removal of the inverse template, the target morphology is achieved. Anodized metal oxides (which will be referred to as AMO from here on), such as anodized aluminum oxide (AAO), are the most commonly used, versatile templates.^26,27^ These templates are fabricated by anodizing the corresponding metal in an electrolyte solution, leading to vertically oriented nanoscopic channels. The pore size and pitch-to-pitch spacing of AAO can be tuned between 10–1000 nm by varying the electrolyte and anodizing voltage (10–450 V).^28^ In another method, block copolymer (BCP) films with pores oriented perpendicular to the substrate serve as inverse templates for vertical nanostructures,^29,30^ from which feature sizes as small as 10–80 nm are obtained. However, both the pore size and pitch-to-pitch spacing is difficult to control in BCP templates.^31^ This limited tunability makes them less versatile and less commonly used than AMO templates. Vertically oriented nanostructures are obtained using both AMO and BCP templates by completely or partially filling their pores with the material of interest, and subsequently removing the template. Either solid nanowires or hollow nanotubes can be obtained by fully or partially filling the nanosized pores.

Despite the generality of these templated approaches, the need for post-synthetic template removal to release the nanostructures grown within pores is undesirable for a number of materials. Strong acids or bases are required to remove AMO templates,^32,33^ whereas harsh etching conditions using oxygen plasma, or dissolution with strong organic solvents, are needed for BCP template removal.^34,35^ Exposing the target electronic materials to such processing conditions can have detrimental effects on their electronic properties, which is especially the case for conducting, π-conjugated polymers or small molecule conductors or semiconductors.^36^ Additionally, for a number of soft materials, the removal of the template can easily cause their vertical structure to distort or collapse under their own weight, diminishing the degree of orientation. Furthermore, AMO templates do not offer a straightforward path for patterning the deposition location of vertically oriented nanostructures. Thus, an alternative template for the growth of a wide-ranging arrays of vertical nanostructures is highly desirable.

**Fig. 1:**
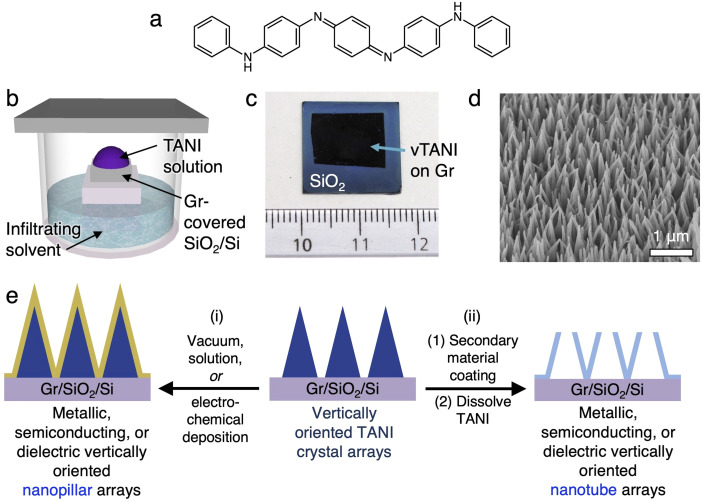
(a) Chemical structure of tetraaniline (TANI). (b) Schematic illustration showing the vTANI crystallization. (c) Photograph and (d) SEM image (45° tilted view) of the vTANI. (e) Schematic diagram illustrating the usage of vTANI as a universal template for vertically oriented nanopillars or nanotubes.

In this work, we explore a different approach: using a direct template that resemble the desired vertically oriented morphology. The material of interest can be coated on the exterior of the existing template, which eliminates the need for template removal. In theory, all existing vertically oriented nanoarrays can serve as direct templates. However, these structures are typically difficult to generate, require multi-step processes, not scalable, or expensive, so it is not desirable to use them as sacrificial templates for other materials. Furthermore, for the coating materials to diffuse throughout the vertical array and uniformly coat the surfaces, the spacing between the vertical nanostructures need to be sufficiently large, which is not straightforward to control. We have previously demonstrated a one-step, solution-based method to grow vertically oriented tetraaniline (TANI) nanopillars by using graphene as the substrate.^37^ The π–π interaction between the aromatic moieties in TANI and graphene provides a strong interface. The high selectivity of TANI nanopillars to grow on graphene also provided a direct path for the patterning of vertical structures. In this work, we demonstrate that these low-cost, scalable, and patternable vertical nanostructures are excellent direct templates for producing vertical orientation for other materials. Both vertically oriented nanopillars and nanotubes (Fig. 1e) are possible, and their size, height, and spacing are tunable. We demonstrate the versatility of this template by employing a number of widely used deposition methods, including vacuum deposition and electrodeposition, to produce vertical structures with a variety of properties—dielectric, semiconducting, conducting, and polymeric. The patterning of these vertical structures at micron resolution is also illustrated.

## 2. Results and discussion

### 2.1. Template generation & conceptual overview

Our previously developed method to grow vertically oriented nanopillar arrays of tetraaniline (TANI)^37^ (Fig. 1a) simply entails dropping a TANI solution onto a graphene-coated substrate and leaving it undisturbed in a closed chamber containing a precipitating solvent for a few hours (Fig. 1b). The TANI growth is highly selective on the graphene-covered areas (Fig. 1c and S2b†). These vertical TANI crystal arrays are hereon abbreviated as vTANI. They display a high degree of vertical orientation and a high uniformity in areal density and height (Fig. 1d and S2†). The vTANI crystals display a uniquely thin, triangular plate shape, likely due to the growth kinetics and preferential growth axis in relation to the graphene substrate. Using the typical crystallization condition described above, the resulting vTANI crystals have an average thickness of ∼20–30 nm, a base width of ∼200–300 nm, pointy tips, and a height of ∼900–1000 nm. These dimensions can be readily tuned by varying the solvation and annealing solvent conditions.^37^ Collectively, the tunability of the vTANI size and density makes vTANI a versatile template for other materials. This template preparation method is scalable, with readily obtainable complete and uniform coverage of vTANI over 1 × 1.5 cm (Fig. 1c), which is thus far limited only by the size of CVD graphene substrates to which we have access. Furthermore, TANI is stable under ambient conditions. The nanopillar morphology is retained over the course of one year when stored in a dark and dry place, illustrating the structural stability of the vTANI template.

The general route for using vTANI arrays as templates for vertically oriented nanopillars (vNPL) and nanotubes (vNT) is depicted in Fig. 1e: (i) a secondary material is coated onto the surface of vTANI arrays using established methods, affording vNPLs with similar vertical alignment (Fig. 1e, left). No template removal is necessary because the vTANI is encased within the coating material of interest. vTANI is insulating in the as-crystallized, undoped state, so it is not expected to affect the electrical properties of the coating material. Alternatively, (ii) vNTs are produced through subsequent steps of coating, cleavage of the tips, and dissolution of the vTANI core using benign polar organic solvents such as ethanol or acetone, which do not affect the coated material (Fig. 1e, right). To illustrate the versatility of the vTANI template, we mainly focus on using two of the most common coating techniques, vacuum deposition and electrodeposition, to produce vNPLs and vNTs for a range of materials. We choose the following materials as representative examples to illustrate the diverse physical properties achievable: silicon dioxide (SiO_2_) as dielectric, germanium (Ge) as semiconductor, aluminium (Al) as metal, indium tin oxide (ITO) as transparent conductor, and poly(3,4-ethylenedioxythiophene) (PEDOT) as polymeric conductor.

### 2.2. Vertical structures through vacuum deposition

**Fig. 2:**
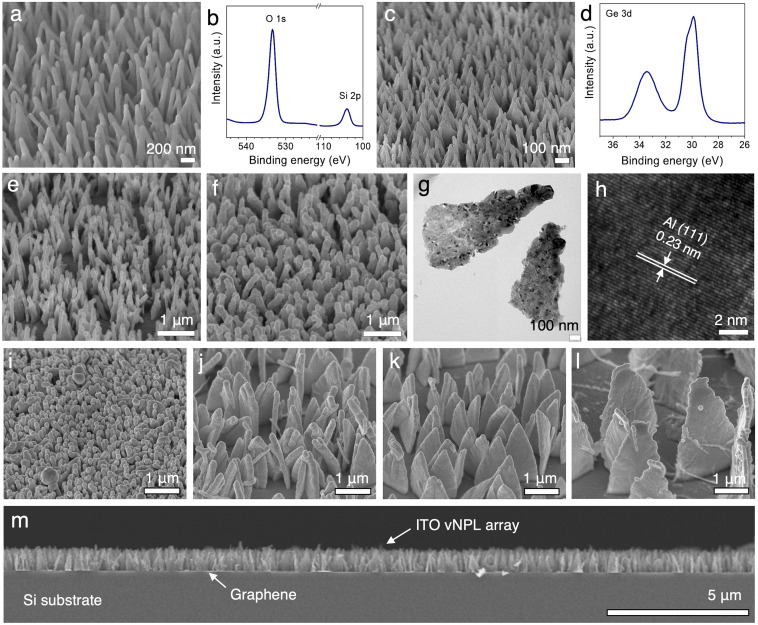
Vertically oriented nanopillar (vNPL) arrays of (a) silicon dioxide, (c) germanium, and (e) aluminum as examples of dielectric materials, semiconductor, and metals, respectively. XPS spectra for the nanopillars in (a) and (c) are shown in (b) and (d), respectively. (e) and (f) Aluminium-covered nanopillars with different coating thicknesses. (g) TEM image and (h) HRTEM image of the nanopillars from (e). (i)–(l) Size and density control of vertically oriented ITO nanopillars. (m) Cross-sectional view of an ITO vNPL array illustrating the high uniformity in height.

Vacuum deposition such as sputtering is one of the most general methods to conformally coat a wide variety of inorganic materials onto a 3D topography. Here, we use sputtering to coat the vTANI templates with SiO_2_, Ge, and Al to created their respective vNPL arrays as representative examples of dielectric, semiconducting, and metallic materials (Fig. 2a, c and e). The chemical nature of these coating materials were confirmed by XPS or high-resolution transmission electron microscopy (HRTEM) (Fig. 2b, d and h). These vNPL arrays exhibit a high degree of uniformity in height across large areas (Fig. 2m).

The coating thickness can be readily controlled by changing the duration of sputtering. For example, the thickness of the Al coating, measured at the vNPL base, can be varied between 30 to 50 nm, while still retaining the vertical orientation and the individual discrete structures (Fig. 2e, f, S3 and S4†). From TEM analysis, the slightly higher contrast near the Al vNPL tips, compared to the bottom, indicates thicker layers of Al are deposited at the top due to unrestricted vapor flow (Fig. 2g). However, the coating covers the entire vTANI, with the polycrystalline Al grains observed across the entire vNPLs (Fig. 2g and h). Such coating gradient is common for vacuum sputtering onto vertical structures, and can often lead the top section to the verge of becoming interconnected.^11^ However, in our case, due to the pointy shape of the vTANI crystals, there is more opening near the top than the bottom, allowing the discrete vNPL morphology to be retained.

Furthermore, the size and density of the vNPLs can be readily controlled by tuning the crystallization conditions of vTANI. As we have demonstrated in our previous work, changing the TANI solution concentration can change the areal density of vTANI arrays.^37^ Changing the solvating solvent can vary the crystal sizes of vTANI in addition to their areal density.^37^ For example, compared to vTANI templates obtained from a 2-propanol solution, using *n*-propanol (*n*PA), tetrahydrofuran (THF), dichloromethane (DCM), and chloroform (CHCl_3_) generates vTANI crystals that are ∼600 nm, 1.5, 2, and 4 μm in height, respectively, with the thickness of the plates confined to the ∼20–80 nm range. The crystal base widths scale with the height difference and average ∼400 nm, 800 nm, 1 μm, and 2 μm for nPA, THF, DCM, and CHCl_3_, respectively, providing stability for the increasing size of the vTANI crystals. This tunability of the feature size and density of vTANI template is passed onto the vNPLs of the target materials. An example using indium tin oxide (ITO), the most widely used transparent electrode material, illustrates the size and density control *via* this general approach (Fig. 2i–l and S6†). ITO interacts with conducting polymers *via* dipolar interaction, creating a stable interface. With a ∼40 nm coating of ITO (*i.e.*, a total ITO thickness of ∼80 nm), arrays of different vNPL sizes and spacings are obtained. In particular, for the dense arrays with small vNPL sizes, individual vNPLs remain discrete due to the larger spacing near vTANI tips (Fig. 2i, S6a and b†). The tall vertical plates remain vertical despite the large height-to-thickness ratio (Fig. 2l, S6g and h†), illustrating the robustness of this general approach. We reason that since no post-deposition template removal is needed, this one-step method for vNPL generation may be particularly beneficial to electronic materials that are sensitive to post-deposition processing conditions.^38,39^

### 2.3. Vertical structures through electrodeposition

While vacuum deposition is a versatile method, it is not applicable to certain classes of materials that need to be solution processed. Conjugated, conducting polymer is an example.^36,40,41^ Due to the electroactive nature of conducting and semiconducting materials, electrodeposition is a widely applicable method for the templated growth (*e.g.*, *via* AMO templates) of their vertically oriented structures.^42,43^ Conformal coatings of desired thickness can be obtained by varying the parameters of electrodeposition such as current, voltage, or scan rate.^44^ To demonstrate the suitability of vTANI templates for creating vertically oriented nanostructures through electrodeposition, we chose poly(3,4-ethylenedioxythiophene) (PEDOT) as the case study material. PEDOT is widely used as an electrode material in organic solar cells, electronics, and bioelectronics.^45,50^

**Fig. 3:**
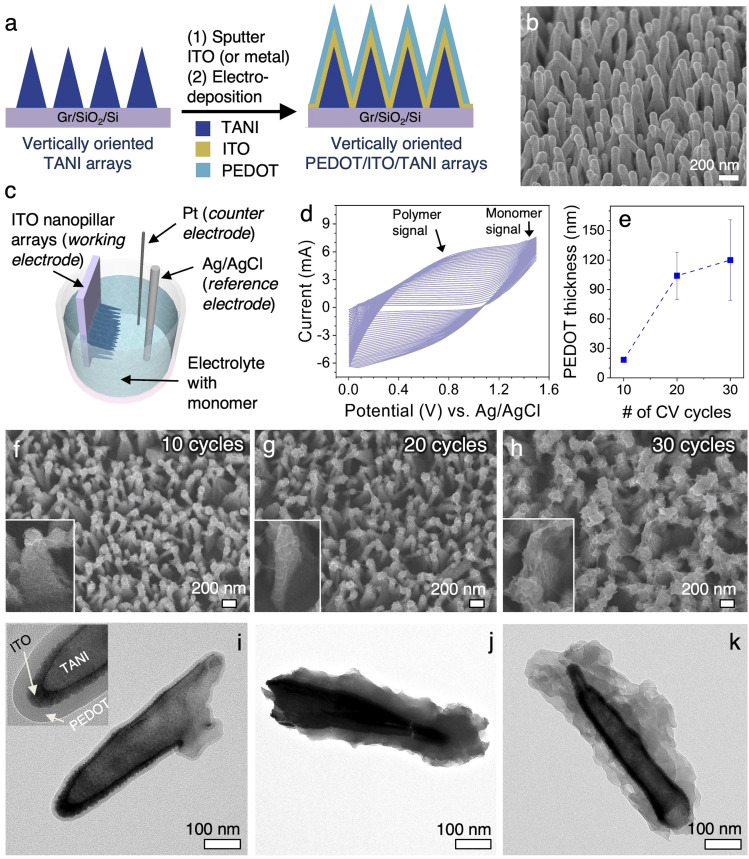
(a) Schematic diagram showing the fabrication process of conformal conducting polymer coating on vertical nanopillar arrays. (b) SEM image of ITO-coated vertical nanopillar arrays. (c) Schematic of the electrodeposition setup. (d) Cyclic voltammetry (CV) of the PEDOT deposition process. (e) Plot showing correlation between PEDOT coating thickness *vs.* CV cycle number. (f)–(h) SEM images showing the vertical nanopillar arrays with PEDOT coating after 10, 20, and 30 cycles of CV, respectively. Magnified images of individual pillars are shown in insets. Their corresponding TEM images are shown in (i)–(k). Inset in (i) illustrates the coaxial PEDOT/ITO/TANI structure of the vertical pillars through a magnified view.

The vTANI template is first coated with ∼10 nm of ITO, converting it into a working electrode for electrodeposition. We chose ITO to mimic the ITO/PEDOT electrode configuration that is the benchmark of organic photovoltaics (Fig. 3a and b).^46^ ITO and PEDOT are known to form stable, high quality interfaces,^11^ allowing ITO to serve as a suitable working electrode for the electrodeposition of PEDOT. Electrodeposition through potentiodynamic conditions *via* cyclic voltammetry (CV) was carried out with 0.1 M EDOT as the monomer, and with 0.1 M tetrabutylammonium hexafluorophosphate (Bu_4_NPF_6_)/acetonitrile solution as the electrolyte (Fig. 3c). The EDOT monomer is electrochemically polymerized into PEDOT, doped by the PF_6_^−^ ions, on the surface of the ITO vNPLs working electrodes by cyclically scanning between 0 and 1.5 V, a value that is beyond the oxidative potential of EDOT (∼1.2 V), for different numbers of cycles. The CV of the electrodeposition process is shown in Fig. 3d. The PEDOT polymer signal at ∼0.75 V increases consistently with increasing numbers of CV cycles, indicative of the increasing amount of PEDOT growth and their electroactive nature, which facilitates the electrodeposition of additional PEDOT.^11^

Fig. 3f–h and 3i–k show the SEM and TEM images, respectively, of the ITO vNPL arrays after 10, 20, and 30 cycles of potentiodynamic electrodeposition of PEDOT. The edge-to-edge inter-pillar spacing for the ITO vNPL arrays is ∼150–200 nm near the top (Fig. 3b). The inter-pillar spacing consistently decrease with increasing CV cycles. A conformal, uniform PEDOT coating of ∼20 nm is obtained with 10 cycles of CV. The vertical pillars remain discrete near the top (Fig. 3f). TEM characterization clearly shows discrete layers within the coaxial structure of vTANI core-ITO shell-PEDOT coating (Fig. 3i, inset). As the number of CV cycles is increased to 20, a thicker PEDOT coating (∼50–80 nm) is obtained, which is accompanied by a small amount of inter-connectedness between adjacent pillars (Fig. 3g and j). With 30 CV cycles, the coating thickness increases further to over 100 nm (Fig. 3k), approaching the inter-pillar spacing of the ITO vNPL electrodes. As a result, some adjacent pillars become interconnected (Fig. 3h). The PEDOT coatings also start to appear rougher, which is presumably due to the low terrace/interlayer diffusion of monomers during polymerization for longer times (more cycles in our case).^47^ The relationship between the number of CV cycles and the average PEDOT coating thickness is summarized in Fig. 3e. Growth rate decreases after ∼20 cycles of CV because the initial layers of PEDOT was deposited onto the highly conductive ITO vNPL electrodes, but the subsequent layers of PEDOT are deposited onto the existing PEDOT coating, which is less conductive than ITO, hence leading to a lower current density and decreased rate of polymerization.^11^

Virtually all CPs can be electrodeposited due to their electroactive nature.^48^ A large variety of metals can also be electrodeposited.^49^ This case study with PEDOT illustrates the potential to coat vTANI templates with other materials *via* electrodeposition.

### 2.4. Vertical structures through spin-coating

For solution-processable materials such as functionalized conjugated polymers or small molecules, spin coating is the most common method to obtain 2D thin films.^50^ However, this approach is generally challenging for forming conformal coatings onto 3D structures because solutions tend to settle, thus forming a dense film at the bottom of the 3D structures, yet leaving the tops uncoated. We tested out the feasibility of creating vNPL arrays *via* spin coating using two benchmark materials for solution-processable organic photovoltaics: poly(3-hexylthiophene) (P3HT) and a functionalized C_60_ derivative, *t*-butylbiphenyl C_60_ (C_60_-TBBP), which are p-type and n-type semiconductors, respectively.^46,50,51^ ITO- and Al-coated vTANI are chosen as the templates to mimic the ITO-P3HT anode and Al–C_60_ cathode contact in organic solar cells.^46^

Spin coating a high concentration of P3HT solution (15 mg mL^−1^ in chloroform) onto ITO-coated vTANI only led to a thin (∼5 nm) coating (Fig. S7†), consistent with the general challenges of coating vertical structures using spin coating. However, the P3HT coating covers the entire pillar, both on top and bottom, likely due to the conical shape of the ITO/vTANI pillars. On the other hand, a considerably thicker coating of C_60_-TBBP (∼15–20 nm) forms on the Al-coated vTANI electrodes by spin coating a 15 mg mL^−1^ solution in *o*-dichlorobenzene (Fig. S8c and d†). This is likely a result of the propensity of the C_60_-TBBP molecules to orient into shuttlecock stack-like structures.^51^ Furthermore, it can be observed on SEM images that the spun C_60_-TBBP coating bridges adjacent vNPL in some places. However, due to the unique conical morphology of the vTANI template, the C_60_-TBBP between the adjacent pillars often form thin sheet-like structures rather than a solid film, creating additional active surface areas (Fig. S8b†).

Collectively, our results indicate that spin coating can be a viable method for coating solution-processable materials onto vTANI templates, but process optimization will be needed.

### 2.5. Patterning of vNPL arrays

**Fig. 4:**
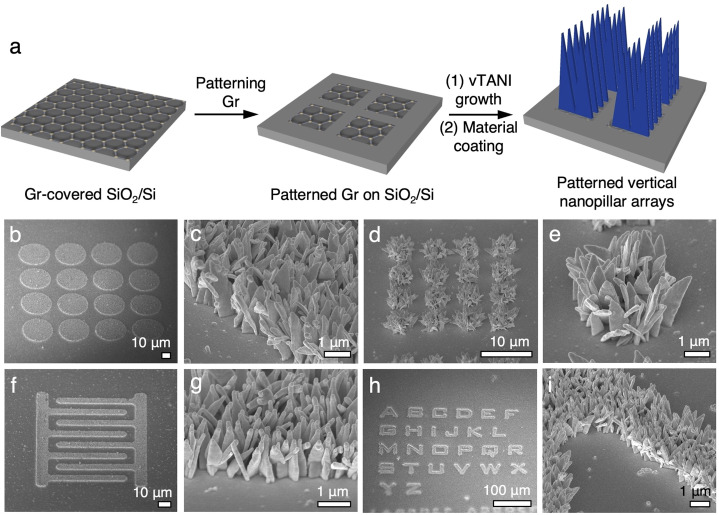
(a) Schematic diagram showing the general patterning flow for vertically oriented ITO nanopillars. (b)–(e) SEM images of various patterns including (b) and (d) circles of different sizes, (f) interdigitated electrodes, and (h) the alphabet. Magnified images of the edges of each pattern in (b), (d), (f) and (h) are shown in (c), (e), (g), and (i), respectively, to illustrate the high resolution of the patterned features.

Our previous work has illustrated that the growth of vTANI is highly selective on graphene.^37^ Hence, patterning the graphene substrate using photolithography prior to vTANI crystallization can lead to the patterning of vTANI arrays. Here, we take advantage of this selectivity to demonstrate the patterning of vNPL for other materials that are templated by vTANI. Following the patterning of graphene and selective vTANI growth, sputtering is used to coat the materials of interest to create patterned vNPL (Fig. 4a). Using sputtered ITO as a model material, patterned vertical arrays of any shape, such as circles, interdigitated electrodes, and the alphabet, can be readily obtained (Fig. 4b–i). The feature resolution of the ITO vNPL arrays is high, with vertical arrays confined to the graphene patterns as small as 3–5 μm in width (Fig. 4d, e, h and i). This feature resolution is currently limited by the resolution of the photolithography process. All the patterns, regardless of sizes, show sharp edges, illustrating the high spatial resolution (Fig. 4b–i).

In contrast to the vTANI templates, patterning of vertical nanopillar arrays is challenging using AMO templates. Furthermore, since the graphene patterning step is carried out before vTANI growth and secondary material coating, the materials of interest will not be subject to harsh solvent or heating conditions that are typically involved with lithography processes.

### 2.6. Template for nanotubes

Vertically oriented nanotubes (vNTs) are attractive architectures for energy storage or catalysis applications due to the doubling of surface area. They can also serve dual functions in bioelectronic applications as nanoneedle arrays for drug delivery while the conductive shells monitor or measure biometric signals.^52–54^ In addition to vertically oriented nanopillars, our vTANI arrays can also serve as templates for the fabrication of vNTs.

**Fig. 5:**
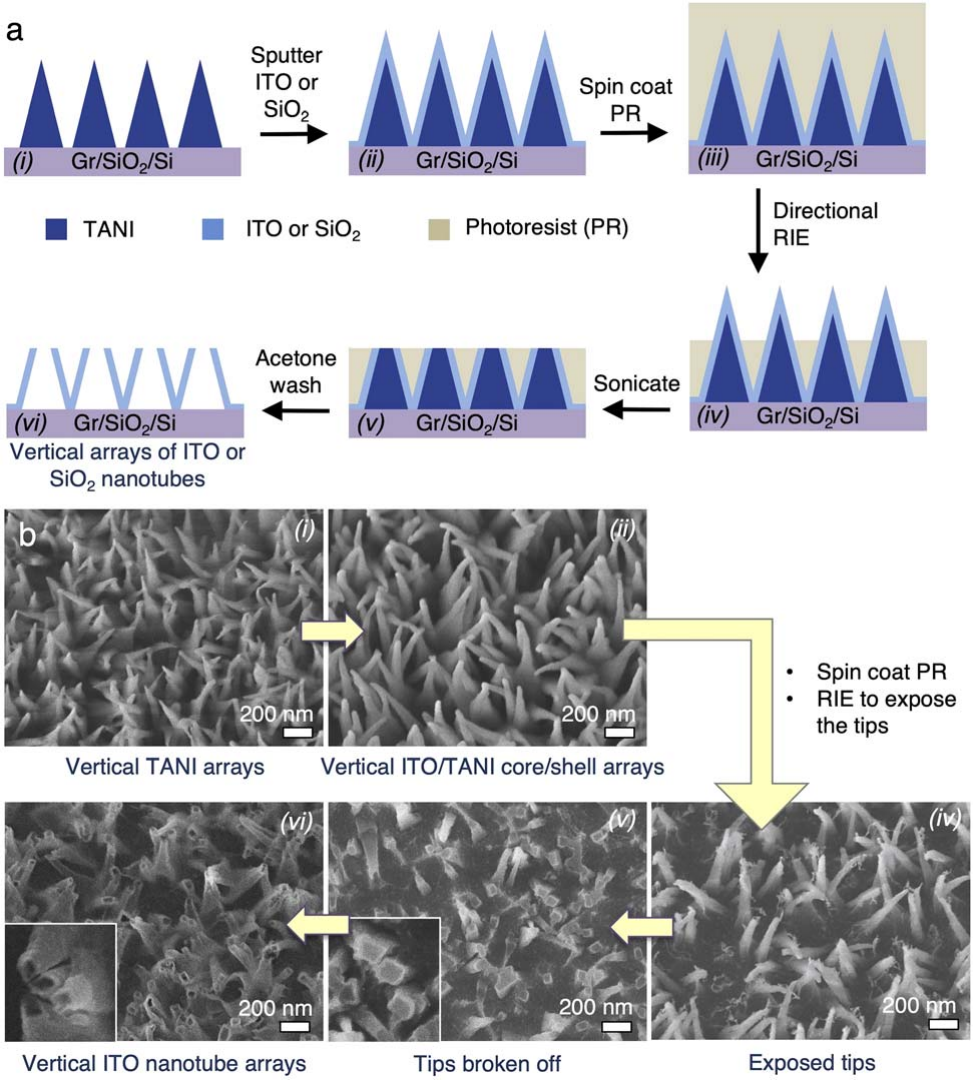
(a) Schematic of the fabrication flow and (b) SEM image series for the fabrication steps of vertical nanotube arrays.

The vTANI cores do not need to be removed when vNPLs are desired. However, due to the high solubility of TANI in polar organic solvents, they can be dissolved away when necessary, leaving behind vNT arrays in a lost-wax-type operation. A typical process is illustrated in Fig. 5a, with the corresponding SEM images for each step arranged in the same order in Fig. 5b. ITO is again used as a model material here, but the same process should be applicable to other coating materials. The ITO vNPLs are first completely covered by a photoresist. The spin coating process of the viscous photoresist appears to tip over a small amount of vNPLs, leading to a slightly reduced vNPL density and resolution (Fig. 5b(ii)–(iv)). Subsequently, the tips of the pillars are exposed through directional, partial reactive ion etching (RIE) of the photoresist (Fig. 5b(iv)). Due to the small diameter of the tips of the vNPLs, prolonged or intense sonication will break off the tips, exposing the vTANI core. The ITO/TANI shell/core structure can be clearly visualized in SEM images (Fig. 5b(v)). Finally, washing with a solvent (*e.g.*, acetone) simultaneously dissolves the TANI core and the remaining photoresist, leading to vertically oriented arrays of nanotubes (vNT). The cavities of the nanotubes can be seen in Fig. 5b(vi).

**Fig. 6:**
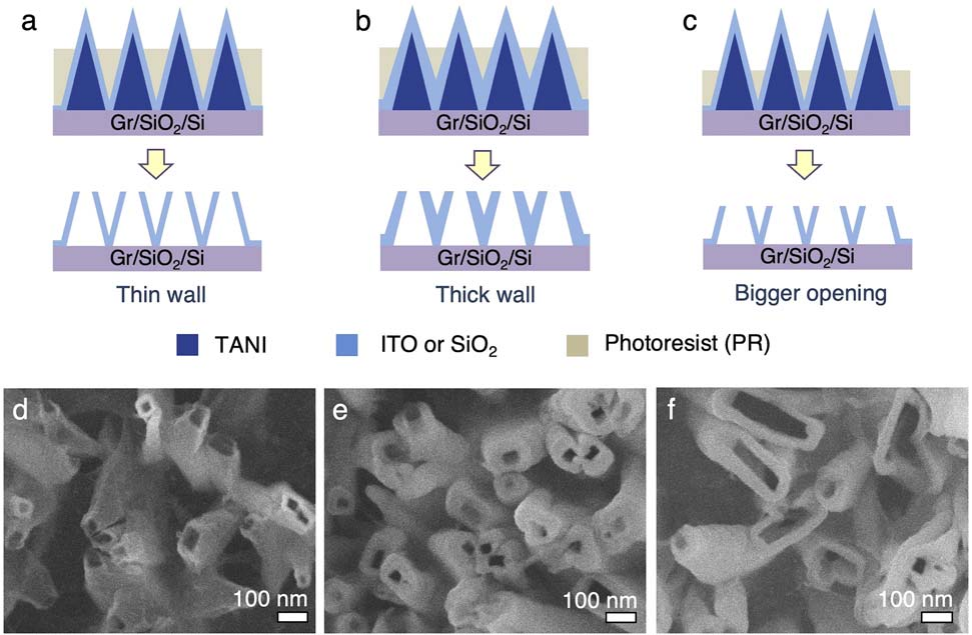
(a)–(c) Schematic diagram illustrating methods for controlling the wall thickness and opening size of the nanotube arrays. (d)–(f) SEM images of the resulting vertical nanotube arrays for (a)–(c), respectively.

Additionally, the wall thickness and opening size of the nanotubes can be readily tuned (Fig. 6a). The wall thickness of the vNT can be controlled by changing the thickness of the coating material (Fig. 6a, d*vs.*6b, e). The size of the nanotube openings can be controlled by the length of the exposed nanopillar tips from the photoresist layer; for example, if a larger tube opening is desired, more photoresist can be directionally etched away to expose a larger cross-sectional area of the nanopillars (Fig. 6c and f). However, the uniformity of the vNT openings decrease with increasing opening size (Fig. 6f). This is likely because the cross-sectional area of the vNPLs increases from the tip to the base. The exposed vNPL tips from the photoresist can be easily broken off under sonication. However, when a larger cross-sectional area of the vNPL is exposed (Fig. 6c), it becomes more difficult to uniformly fracture them at the photoresist layer edge. Some vNPLs appear to break at the tip, leading to a distribution of opening sizes (Fig. 6f). We reason that the size, density, and patterns of the vNT arrays can also be potentially tuned by applying the strategies discussed for vNPL (Fig. 2i–l and 4).

## 3. Conclusion and outlook

We have demonstrated a new direct template for creating vertically oriented structures, which is based on the vertically oriented morphology of TANI nanocrystals (vTANI) grown on a graphene substrate. These nanocrystal templates exhibit the following attributes: (1) versatility in material composition and deposition methods. (2) Both vertically oriented solid vNPLs and hollow vNTs can be generated. (3) For vNPLs, no post-deposition template removal is needed. (4) The size, density, and deposition location of these vertical arrays can be readily controlled.

Looking at the future, the vTANI templates can potentially become more accessible by using mechanically exfoliated graphene. Preliminary testing reveals that vTANI growth is selective on peeled graphene flakes, and the vertical growth direction is also retained (Fig. S9†). The process can also be scaled up by exploiting large-area transfer approaches for CVD graphene.^55^ In addition, with graphene being an excellent barrier material to solvents, multi-layer vertical structures are possible. Preliminary testing shows that when graphene is transferred on top of a GaAs vertical nanopillar array, vTANI can be selectively grown on the graphene while leaving the GaAs area intact (Fig. S10†). It illustrates the potential of using this templated approach to create multi-layer, vertical, heterojunction electronic devices.

## 4. Experimental

### 4.1. Materials and vTANI template generation

Phenyl/phenyl-capped tetraaniline (TANI) was synthesized *via* a single-step condensation reaction following a previously reported route.^56^ CVD graphene was grown and wet transferred onto silicon wafers using polylactic acid as previously reported.^57,58^ vTANI crystallization was performed using a previously developed solvent infiltration method (Fig. 1b).^37^ Fundamental characterization of vTANI and graphene can be found in our previous work.^37^ EDOT, Bu_4_NPF_6_, acetonitrile, and P3HT were purchased from Sigma-Aldrich. C_60_-TBBP was synthesized using a previously reported method.^51^

### 4.2. Inorganic nanowires

Aluminum (Al), germanium (Ge), silicon dioxide (SiO_2_), and indium tin oxide (ITO) sputter sources were obtained from Kurt J. Lesker. These substances were deposited by RF magnetron sputtering using a Denton CVC-601 sputter at room temperature, with a 5 mT deposition pressure and 30 sccm of argon gas. The deposition rate for Al, Ge, SiO_2_, and ITO were 11.9, 12.5, 3.1, and 9.3 nm min^−1^, respectively.

### 4.3. Spin coating

Both P3HT and C_60_-TBBP solutions were spin coated using a gradient spinning method, with the spinning speed incrementally increased from 30 s at 1000 rpm, 30 s at 1500 rpm, and 30 s at 2000 rpm.

### 4.4. Electrodeposition

Electropolymerization of PEDOT (Fig. 3c) was carried out under potentiodynamic conditions with the potential applied by a VersaSTAT 3-400 potentiostat/galvanostat (Princeton Applied Research) using a standard one compartment, three-electrode setup. The ITO nanopillars served as the working electrode, Pt wire as the counter electrode, and Ag/AgCl as the reference electrode. The electrolyte was comprised of 0.1 M EDOT dissolved in a 0.1 M tetrabutylammonium hexafluorophosphate (Bu_4_NPF_6_)/acetonitrile solution.

### 4.5. Patterning

Microscopic patterning of graphene was carried out using standard photolithography procedures. In brief: the graphene-covered SiO_2_/Si substrates were covered by AZ5214 photoresist (MicroChemicals GmbH) by spin coating at 3500 rpm. A photomask with desired patterns were aligned on top with a Karl Suss MA6 mask aligner followed by 6 s of UV exposure at 8 mW. After removing the non-irradiated photoresist using AZ Developer, the exposed graphene was removed by O_2_ plasma etching at 100 W for 60 s. The remaining photoresist was then striped using acetone followed by a 1 hour baking step at 450 °C under Ar protection to thoroughly remove the remaining photoresist residues. vTANI was then selectively grown on the patterned graphene, followed by sputtering of desired inorganic materials *via* vacuum deposition.

### 4.6. Nanotube fabrication

For the nanotube fabrication (Fig. 5 and 6), the ITO-coated vTANI were embedded in AZ5214 photoresist (MicroChemicals GmbH) *via* spin coating. An optical microscope was used to examine the piece to ensure the arrays were completely embedded in the photoresist. Directional etching of the photoresist was carried out using a Technics Micro-RIE Series 800 Plasma System at 1 min intervals. SEM was used to examine the sample after each minute of etching until the desired height of tips was exposed. The sample was then sonicated in a bath sonicator for 4–5 min to break off the exposed tips. Finally, the entire wafer was soaked in heated acetone (∼50 °C) for 40 min with intermittent agitation to dissolve both the remaining photoresist and the TANI core.

### 4.7. Characterization

Scanning electron microscopy (SEM) images were collected on either JEOL JSM-6700 SEM or Zeiss Gemini 500 SEM. Transmission electron microscopy (TEM) samples were prepared by gently rubbing a TEM grid against the vertical nanostructure arrays, which facilitated the transfer of some nanostructures onto the TEM grid surface. TEM images and SAED patterns were collected on either a FEI/PHILIPS CM 120 TEM or a Talos F200C G2 TEM at an accelerating voltage of 200 kV. Au standards were used to calibrate the TEMs for HRTEM. XPS analysis were carried out using a Nexus X-ray Photoelectron Spectrometer.

## Author contributions

B. X. carried out fabrication and characterization. D. W. performed characterization. I. M. H., M. H. and Y. R. designed and synthesized materials. D. W. and Y. W. drafted the manuscript. Y. W. and Y. R. supervised the project. All authors contributed to data analysis and manuscript preparation.

## Conflicts of interest

There are no conflicts to declare.

## Supplementary Material

NA-005-D3NA00476G-s001
