# Supplementary material for: A new and versatile template towards vertically oriented nanopillars and nanotubes
Source: Nanoscale Adv. 2023 Aug 9;5(17):4489–98. doi: 10.1039/d3na00476g (PMC10448359; doi:10.1039/d3na00476g)
Supplement: NA-005-D3NA00476G-s001 [file NA-005-D3NA00476G-s001.pdf]

Electronic Supplementary Material (ESI) for Nanoscale Advances.
This journal is © The Royal Society of Chemistry 2023

Supplementary Information

TANI characterization

The UV-Vis-NIR spectroscopy of our Ph/Ph TANI is shown in Fig. S1a. The λ_{1max} at 309 nm and the λ_{2max} 570 nm are consistent with previous reported values on this molecule in the emeraldine base (EB) form, which indicates that our Ph/Ph TANI has the same chemical structure and oxidation state. Additionally, the symmetric shape of the peaks is an indication that our product is pure.[1]

The chemical identity of phenyl/phenyl-capped tetraaniline (Ph/Ph TANI) oligomer can be confirmed by FT-IR. The spectrum (Fig. S1b) contains many of the characteristic features found in polyaniline, as well as those found in phenyl/amine-capped TANI.[2,3,4] One of the notable features in the FT-IR spectra of polyline and other aniline oligomers is the asymmetric N-H stretching. This peak appears at 3384 cm^{-1} for our Ph/Ph TANI, but is a relatively weak signal, which is likely obscured by the broad O-H stretching from trace water in the sample. Many other characteristic TANI signals are present in the spectrum, including the C-N stretching of the secondary amines at 1308 cm^{-1}, the aromatic C-H stretching at 3043 cm^{-1}, the aromatic C-H out-of-plane bending at 803 and 740 cm^{-1}, as well as signals characteristic of TANI emeraldine base, including the benzoid C=C stretching at 1488 cm^{-1}, and the quinoid C=C stretching at 1573 cm^{-1}.

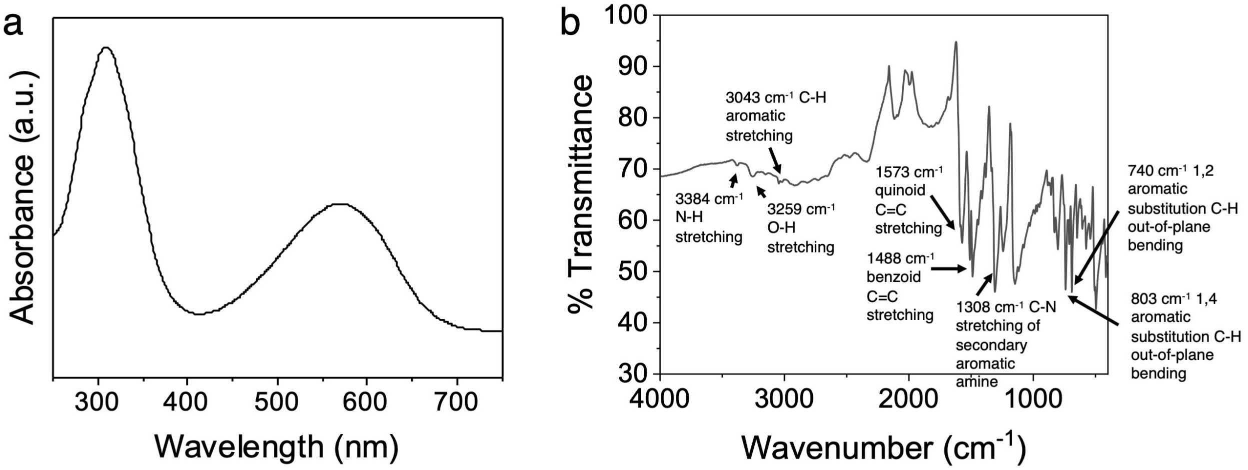

Figure S1. (a) UV-vis and (b) FT-IR spectra of phenyl/phenyl-capped TANI.

vTANI template generation procedure:

In a typical procedure, a crystallization chamber is created by partially filling a glass Petri dish with n-hexane, a nonsolvent to TANI (Fig. 1b). A piece of graphene-coated SiO_2/Si wafer is raised above the liquid level with a thick glass stage. A 2 mg/mL TANI/2-propanol solution is then dropped onto the wafer, and the Petri dish is closed, but not sealed. Vapor of the nonsolvent, n-hexane, slowly diffuse into the TANI solution, decreasing the TANI solubility, leading to supersaturation followed by the selective nucleation and crystallization on the graphene-covered area due to the strong π-π interaction between the TANI and graphene. Select area electron diffraction (SAED) shows that each vertical TANI structure is single crystalline in nature. The TANI growth is highly selective on the graphene-covered areas, illustrated by the sharp interfaces (Fig. S2a, b). They display a high degree of vertical orientation and a high uniformity in areal density and height (Fig. S2c, e-g).

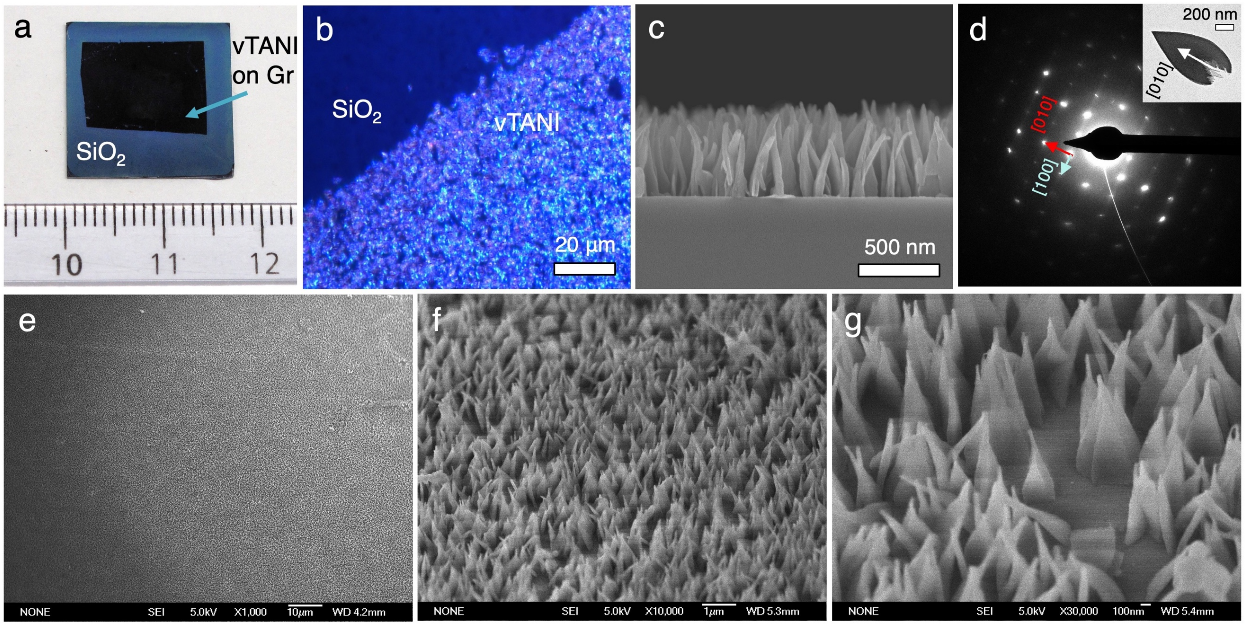

Figure S2. (a) Photograph and (b) optical microscope image showing the highly selective growth of vTANI on the graphene-covered areas on the SiO_2/Si substrate. (c) Cross-sectional SEM image showing the vertical orientation and height uniformity of vTANI. (d) SAED pattern showing the single crystalline nature of vTANI. (e)-(g) SEM images of vTANI arrays under various magnifications viewed at a 45° tilting angle.

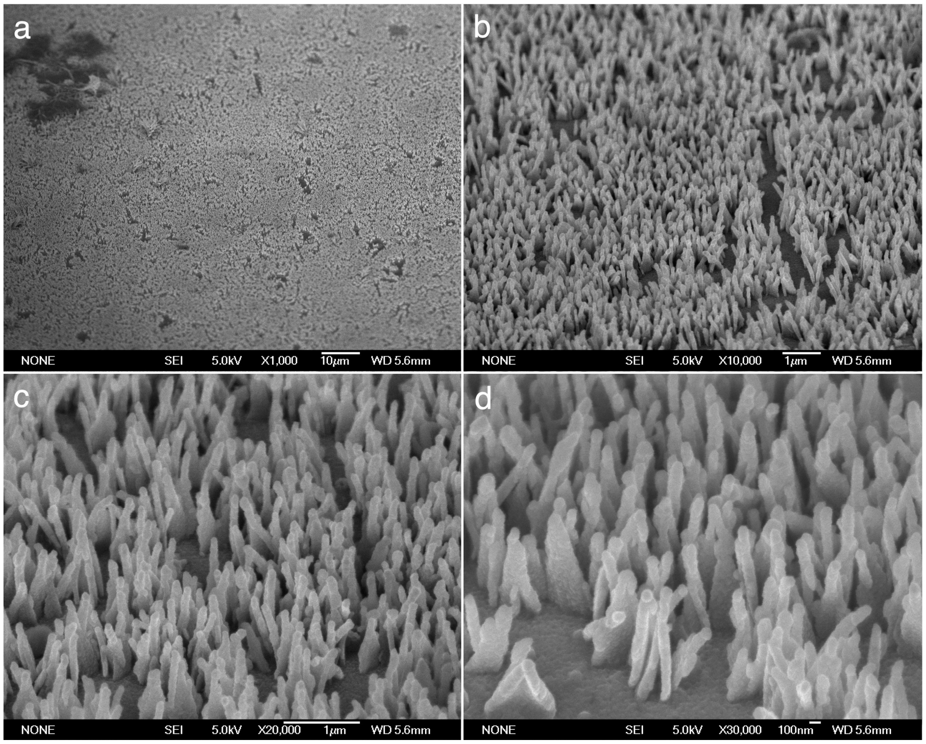

Figure S3. (a)-(d) SEM images of vTANI arrays coated with ~30 nm of Al (i.e., leading to a total plate thickness of ~80 nm) under various magnifications viewed at a 45º tilting angle.

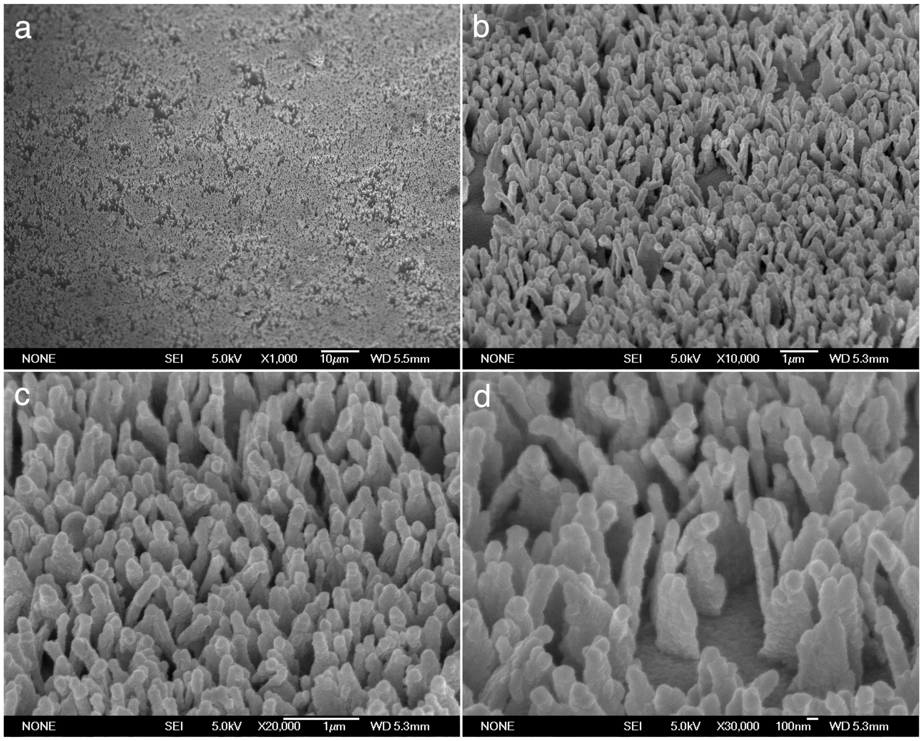

Figure S4. SEM images of vTANI arrays coated with ~50 nm of Al (i.e., leading to a total plate thickness of ~120 nm) under various magnifications viewed at a 45º tilting angle.

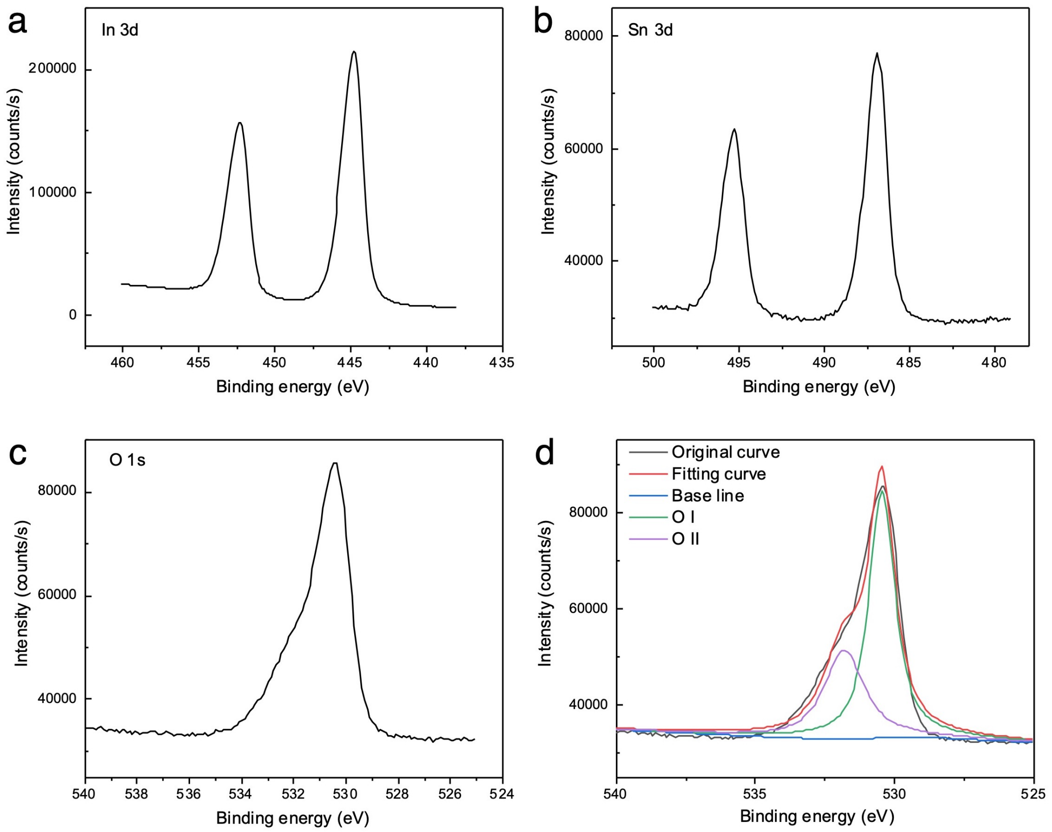

Figure S5. XPS of vTANI coated with ITO.

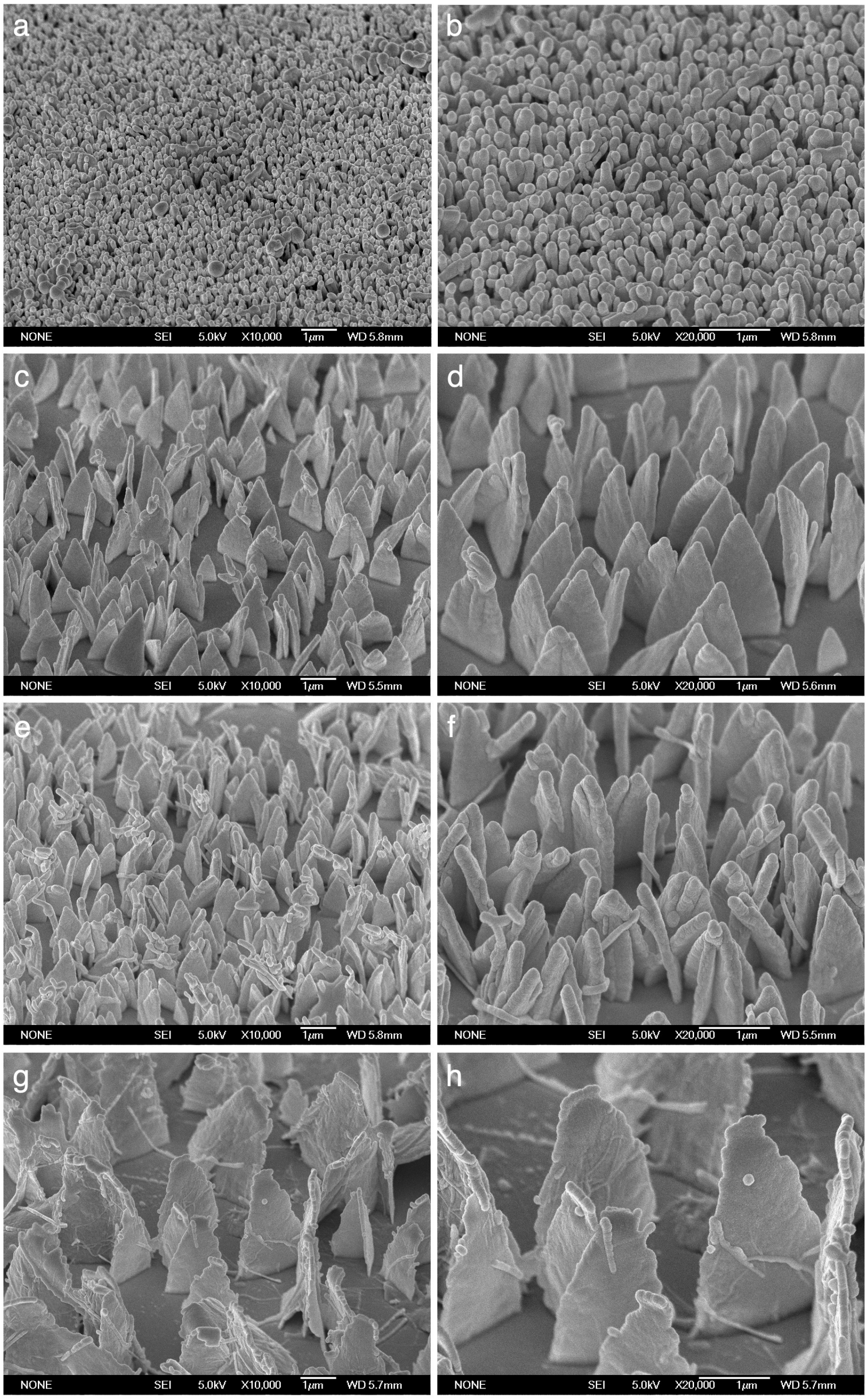

Figure S6. SEM images of arrays of Al-coated vTANI with different pillar sizes and densities viewed at a 45º tilting angle. Solvents used for growing the vTANI in (a)-(b) is 2-proponol, in (c)-(d) is THF, in (e)-(f) is dichloromethane, and in (g)-(h) is chloroform.

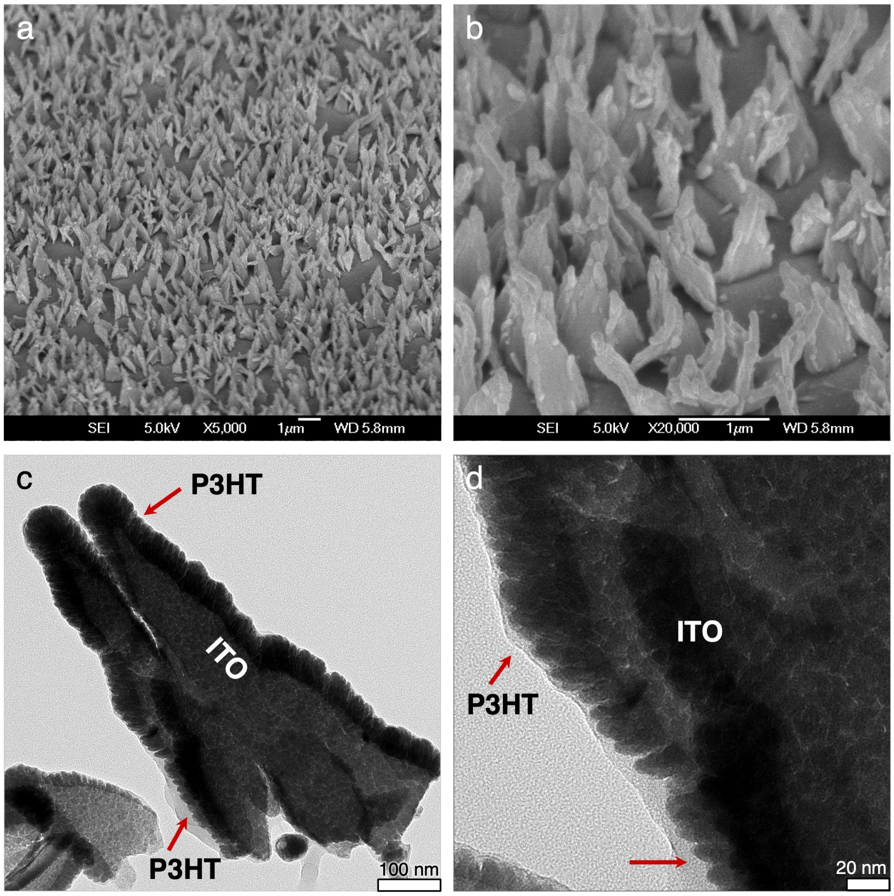

Figure S7. (a)-(b) SEM and (c)-(d) TEM images of ITO vNPL coated with spin-coated P3HT (15 mg/mL in *o*-dichlorobenzene). Arrows on (c) and (d) highlight thin P3HT coating.

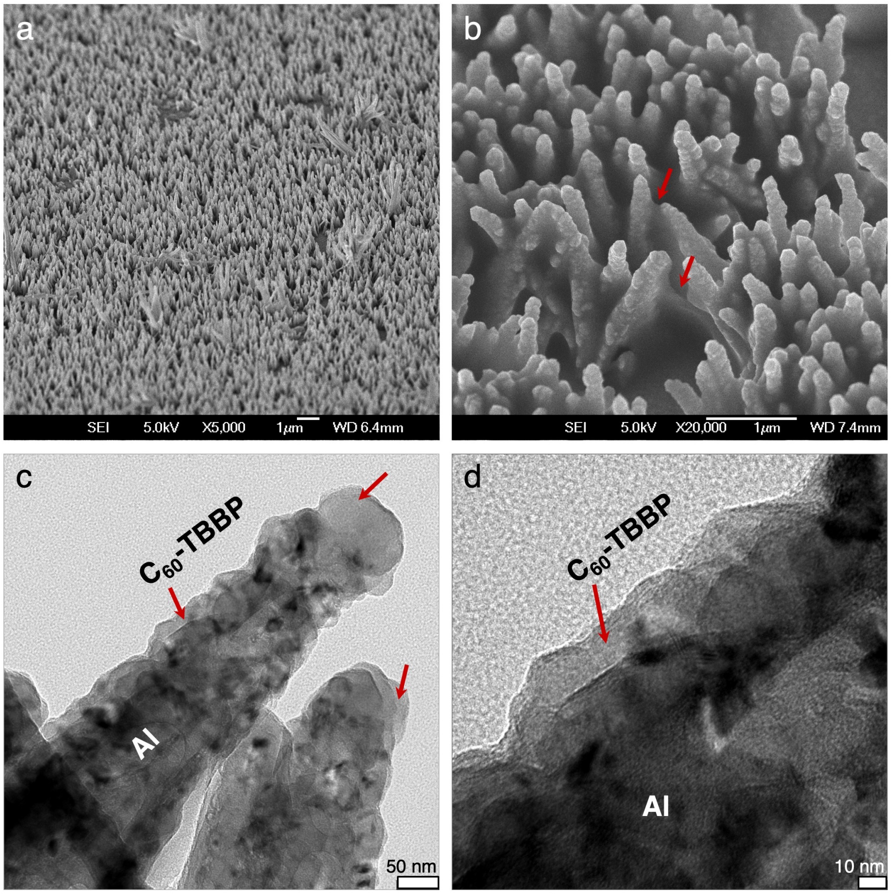

Figure S8. (a)-(b) SEM and (c)-(d) TEM images of Al vNPL coated with spin-coated C_{60}-TBBP (15 mg/mL in chloroform). The arrows on (b) indicate areas where nanosheets of C_{60}-TBBP are formed due to the conical and plate-like geometry of the Al vNPL. Arrows on (c) and (d) highlight the C_{60}-TBBP coating.

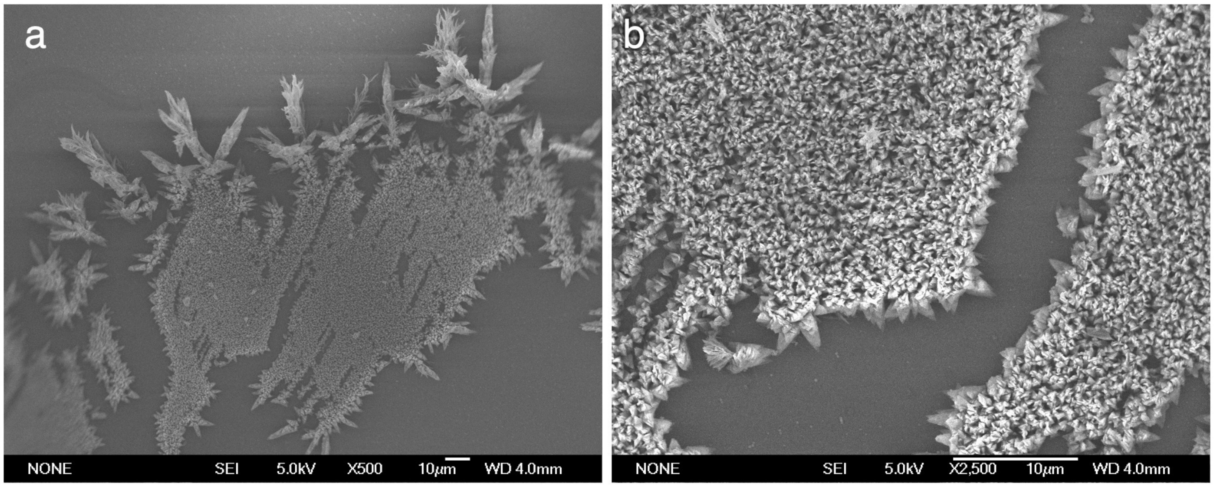

Figure S9. SEM images of vTANI selectively grown on mechanically exfoliated graphene, showing the potential for generating vTANI template through lower cost alternatives.

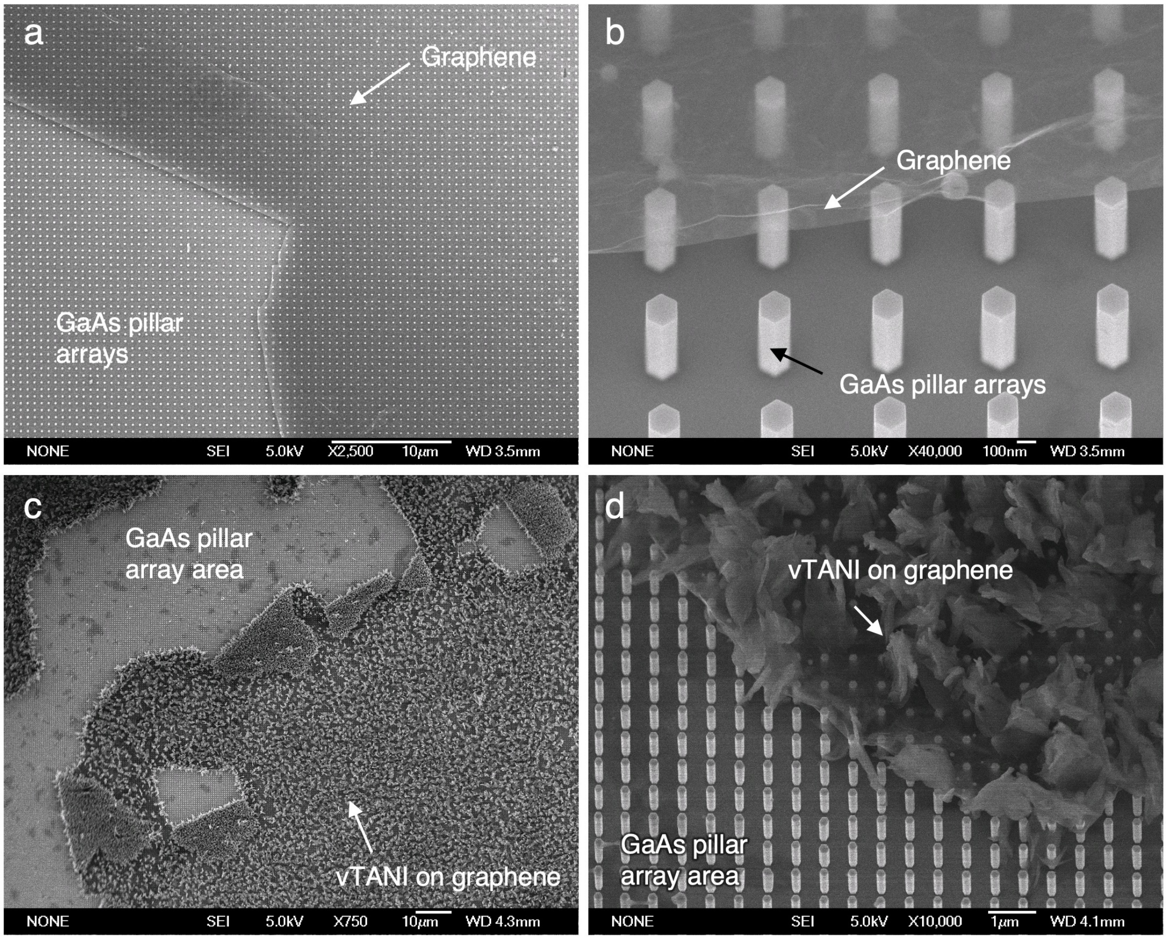

Figure S10. Illustration of the potential of the method described in this manuscript for potentially growing multi-layer structures. (a)-(b) Tilted SEM images of graphene-covered GaAs nanopillar arrays grown by MOCVD. (b) offers a magnified view of the edge of the graphene levitated on the GaAs nanopillar arrays. (c)-(d) Tilted SEM images showing the vTANI selectively grown on the graphene that is on top of the GaAs nanopillar arrays, and leaving everything else intact.

References

1. Shao, Z., Rannou, P., Sadki, S., Fey, N., Lindsay, D. M., Faul, C. F., Delineating Poly(Aniline) Redox Chemistry by Using Tailored Oligo(Aryleneamine)s: Towards Oligo(Aniline)-Based Organic Semiconductors with Tunable Optoelectronic Properties. *Chem. Eur. J.*, **17**, 12512-12521 (2011).

2. Zujovic, Z. D., Wang, Y., Bowmaker, G. A., Structure of Ultralong Polyaniline Nanofibers Using Initiators. *Macromolecules* **44,** 2735–2742 (2011).

3. Boyer, M. I., Quillard, S., Louarn, G., Buisson, J. P., Monkman, A., Lefrant, S., Vibrational Analysis of Polyaniline: A Model Compound Approach. *J. Phys. Chem. B* 1998, **102**, 7382–7392 (1998).

4. Wang, Y., Tran, H. D., Kaner, R. B., Template-Free Growth of Aligned Bundles of Conducting Polymer Nanowires. *J. Phys. Chem. C*, **113**, 10346–10349 (2009).
